# The Role of Radiofrequency Hyperthermia in The Radiosensitization of A Human Prostate Cancer Cell Line 

**DOI:** 10.22074/cellj.2017.4460

**Published:** 2017-05-17

**Authors:** Azam Janati Esfahani, Seied Rabi Mahdavi, Mohammad Bagher Shiran, Samideh Khoei

**Affiliations:** 1 Department of Medical Physics, Iran University of Medical Sciences, Tehran, Iran; 2 Radiation Biology Research Center, Department of Medical Physics, School of Medicine, Iran University of Medical Sciences, Tehran, Iran

**Keywords:** Radiation, Hyperthermia, DNA Damage, Apoptosis, Prostate Cancer

## Abstract

**Objective:**

This study evaluated enhanced induced DNA damages and apoptosis of a spheroid culture of DU145 prostate cancer cells treated by a combination of radiofrequency hyperthermia (RF HT) with radiation treatment (RT) from an external radiotherapy machine compared to RT alone.

**Materials and Methods:**

In this experimental study, DU145 cells were cultured as spheroids until they reached 300 µm in diameter. We exposed these cultures to either: RF HT for 90 minutes at 43˚C originated from a Celsius TCS system, RF HT followed by RT at doses of 2 Gy or 4 Gy (15 MV energy) with 15-minute interval, or RT alone at the above mentioned doses. The trypan blue exclusion assay, alkaline comet assay, and annexin V/PI flow cytometry were performed to measure cell viability, the amount of DNA damage in an individual cell as the tail moment, and percentage of induced cell apoptosis in response to treatments explained.

**Results:**

We calculated the thermal enhancement factor (TEF) for the combined treatment regime. RF HT followed by the 4 Gy dose of RT resulted in minimum viability (85.33 ± 1.30%), the highest tail moment (1.98 ± 0.18), and highest percentage of apoptotic cells (64.48 ± 3.40%) compared to the other treatments. The results of the TEF assay were 2.54 from the comet assay and 2.33 according to flow cytometry.

**Conclusion:**

The present data suggest that combined treatment of mega voltage X-rays and RF HT can result in significant radiosensitization of prostate cancer cells.

## Introduction

Radiofrequency (RF) capacitive heating, in combination with radiotherapy has been applied by several investigators as cancer treatment, including deep seated tumors (1,3). Because of the capacitive effects in the tissue, heat is generated (4). It is possible to efficiently heat deeper regions by the application of two electrodes in terms of size, output, and cooling temperature on both sides of the target area as a dielectric material. Hyperthermia (HT) is defined as a therapeutic procedure used to raise an entire body or local tissue temperature to about 42-45˚C (mild HT). Tumor cells most resistant to radiation, including oxygen- and nutrition-deprived cells at an acidic pH and in S-phase, are recognized as the most sensitive to HT (5,6). However, the exact underlying mechanisms of HT have not been determined at the molecular level. It was found that HT in conjunction with radiation had a radiosensitizing effect. Combined HT with radiotherapy in some solid tumors resulted in significant response, better tumor control, and improved overall survival rates (7). 

The use of HT in prostate cancer treatment is controversial because it prevents androgen receptor expression in human prostate cancer cells; hence, they do not respond to hormone therapy (8). DU145 cell line has been obtained from brain metastasis that originated from a prostate adenocarcinoma (9). DU145 is androgen independent (10); therefore, this cell line can be selected for experiments to study the effect of HT. DU145 has the potential for self-assembly and generates three-dimensional multicellular spheroids similar to a tumor architecture with a non-uniform oxygen and nutrient cells (11). Review articles show that a need exists to study the radiosensitizing effect of RF HT on prostate cancer cells. The effect of RF capacitive HT that uses a 13.56 MHz frequency combined with mega voltage X-rays on the human prostate cancer cell line DU145 has not been investigated. 

In the current study, we aimed to evaluate the radiosensitivity of 300 µm DU145 spheroids by the application of RF HT followed by irradiation with 15 MV photons. In order to achieve this goal, we conducted the following tests: i.Viability test by trypan blue; ii. An alkaline comet assay as a genotoxicity test to compare the DNA damages of the treatments; and iii. Flow cytometry annexin V/PI analysis to compare the percentage of apoptotic and necrotic cells created by treatments. We measured the thermal enhancement factor (TEF) after every combined treatment so that it could be considered as an additive effect of HT to radiation, compared to radiation alone. 

## Materials and Methods

### Cell line

In this experimental study, the human prostate carcinoma cell line, DU145, was provided by Pasteur Institute of Iran and maintained in Roswell Park Memorial Institute medium (RPMI-1640, Gibco, NY, USA) supplemented with 10% heat-activated fetal bovine serum (FBS, Gibco, NY, USA), 100 U/ml of penicillin and 100 mg/ml of streptomycin (Biowest, Nuaille, France). DU145 cells were cultured as a monolayer at a density of 10^4^ cells/cm^2^ in T-25 tissue culture flasks (Nunc, Roskilde, Denmark). The cultures were maintained at 37˚C in a humidified atmosphere of 5% CO_2_ and propagated by trypsinization with 1 mM EDTA/0.25% trypsin (w/v) in phosphate- buffered saline (PBS, Sigma-Aldrich, MO, USA). Cells that grew exponentially with an apparent doubling time of 36 hours were used in this study. 

### Spheroid culture

Spheroids were initiated according to the liquid overlay technique (12). A total of 5×10^5^ cells were seeded in 100 mm petri dishes (Jet Biofil Co., Ltd., China) coated with a thin layer of 1% agar (Sigma-Aldrich, MO, USA) with 10 ml of RPMI 1640 supplemented with 10% FBS. The plates were incubated at 37˚C in a humidified atmosphere of 5% CO_2_ . Half of the culture medium was replaced with a fresh medium twice per week. Spheroids grew exponentially with an apparent volume doubling time of 4.4 days. When spheroids reached 300 µm in diameter, they were transferred to a sterile T-12.5 flask (Jet Biofil Co., Ltd., China) filled with RPMI 1640 medium for subsequent exposure to radiation, heat, or the combination of both. 

### Radiation treatment

The T-12.5 flasks (Jet Biofil Co., Ltd., China) that contained 300 µm spheroids and 35 ml RPMI 1640 medium were sealed with Parafilm. For all radiation treatments, we placed the prepared flasks in the center of a water phantom (dimensions: 30×30×15 cm) after which they were exposed to 15 MV X-rays at doses of either 2 or 4 Gy. We used a Linac source (Siemens Primus) for sample irradiation. The third (control) sample did not receive any radiation (0 Gy). 

### Radiofrequency hyperthermia of spheroid cultures

**Fig.1:**
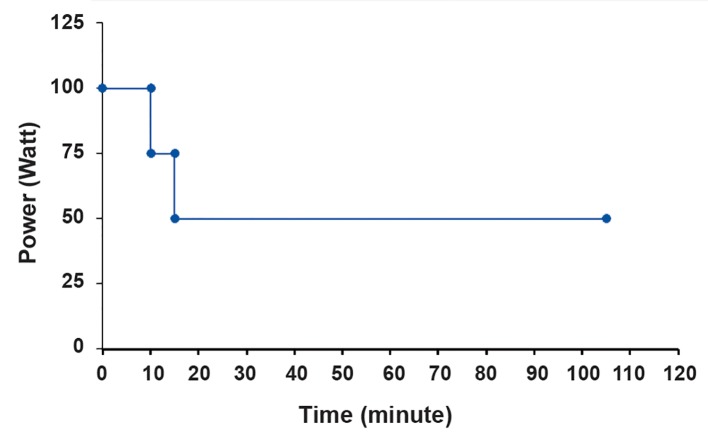
Protocol used to create radiofrequency hyperthermia (RF HT) at 43˚C for 90 minutes.

**Fig.2:**
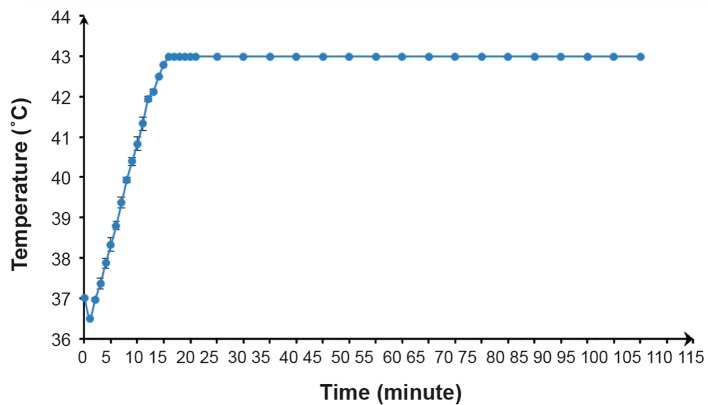
Temperature elevations over time created according to the radiofrequency hyperthermia (RF HT) protocol.

A RF-capacitive HT system, Celsius TCS (Celsius42+, GmbH Company, Cologne, Germany) that operated at a frequency of 13.56 MHz delivered the RF HT treatments at 43˚C 88 Prostate Cancer HT Radiosensitization for 90 minutes. The size of both electrodes was 25 cm in diameter. A T-12.5 flask that contained the spheroids and RPMI 1640 was covered by a towel placed between two electrodes of a device as the dielectric material. We used a step-down power protocol (Fig.1) where the temperature on the central axis of the flask increased after 15 minutes until it reached 43˚C. We did not add this differential time to the heating time. The heating time of 90 minutes indicated that the temperature inside the flask remained at 43˚C ± 0.1 for the entire time (Fig.2). During this protocol, we observed a total energy release of 354.2 kJ. Cells exposed to a temperature of 37˚C served as the control. We used a datalogging thermometer (Extech 421509) that consisted of Teflon-coated thermocouples to record the temperature in the center of the flask. A thermocouple inserted through a plastic catheter was placed in the flask. The thermocouples were calibrated against a standard mercury thermometer. We measured temperatures during RF HT. The accuracy of the thermometer was ± 0.05˚C. 

### Combination therapy: radiofrequency hyperthermia+radiotherapy 

We irradiated the samples at 2 or 4 Gy doses of 15 MV X-rays after 90 minutes of RF HT. The interval between the end of RF HT and the initiation of radiation exposure was fixed at 15 minutes. TEF was the radiosensitizing effect of heat determined as the ratio of the response of cells to RF HT+RT to RT alone at the same dose of radiation. 

### Trypan blue exclusion assay

We mixed a suspension of treated and control single cells from spheroid cultures with trypan blue at a 9:1 ratio. After 3-5 minutes, this mixture was evaluated under a light microscope (Bell, INV- 100-FL) where blue-colored cells were considered nonviable. The ratio of unstained cells to total number of cells was reported as the viability percentage for each cell category. 

### Alkaline comet assay

DNA damage caused by the treatments under study were determined by the alkaline comet assay, according to Fazeli et al. (13). We have measured fluorescence intensity according to CometScore software to quantify the amount of DNA damage as an increase in tail moment, which is the product of the amount of DNA (fluorescence) in the tail and the distance between the means of the head and tail fluorescence distributions. 

### Flow cytometry annexin V/PI assay

The percentage of apoptotic and necrotic cells after treatments were detected by flow cytometry using an annexin V/PI kit (Immunostep Co., Ltd., Spain). According to the manufacturer’s protocol, cells were washed with PBS. Then, 5×10^5^ cells/ml were resuspended with 0.5 ml of 1X Annexin Binding Buffer, after which 5 µl of annexin V-FITC and 5 µl of PI were added to each 100 µl of cell suspension. After 15 minutes, flow cytometry analysis was performed with a BD FACSCalibur (BD Biosciences, USA). We divided the cells into four groups according to annexin/PI results: living cells (annexin V-negative, PI-negative) located in the lower left quadrant of the dot plot; early apoptotic cells (annexin V-positive, PI-negative) located in the lower right quadrant of the dot plot, late apoptotic cells (annexin V-positive, PI-positive) located in the upper right quadrant of the dot plot, and necrotic cells (annexin V-negative, PI-positive) in the upper left quadrant of the dot plot. We assumed that the sum of cells in early and late apoptosis were apoptotic cells. Necrotic cells were designated as the sum of those cells located in the upper left and right quadrants of the dot plot. 

DU145 cells were cultured as spheroids by using the liquid overlay method. When spheroids reached 300 µm in diameter (Fig.1), they were exposed to RT, RF HT, or the combination of both. After treatments, spheroids were treated with 300 µl of 1 mM EDTA/0.25% trypsin (w/v) in PBS for 10 minutes at 37˚C. Trypsin was neutralized by the addition of 700 µl of culture medium that contained 10% FBS. The single cells were counted and tested one hour after treatment by viability, the alkaline comet assay, and flow cytometry. 

### Statistical analysis

Each experiment was conducted in triplicate. Data were presented as mean ± SEM. Statistical analysis was performed using one-way analysis of variance (ANOVA) followed by Tukey’s test as the post-hoc analysis using SPSS version 17. P<0.05 compared to the control group were considered statistically significant. 

## Results

### Effect of treatments on cell viability

**Fig.3:**
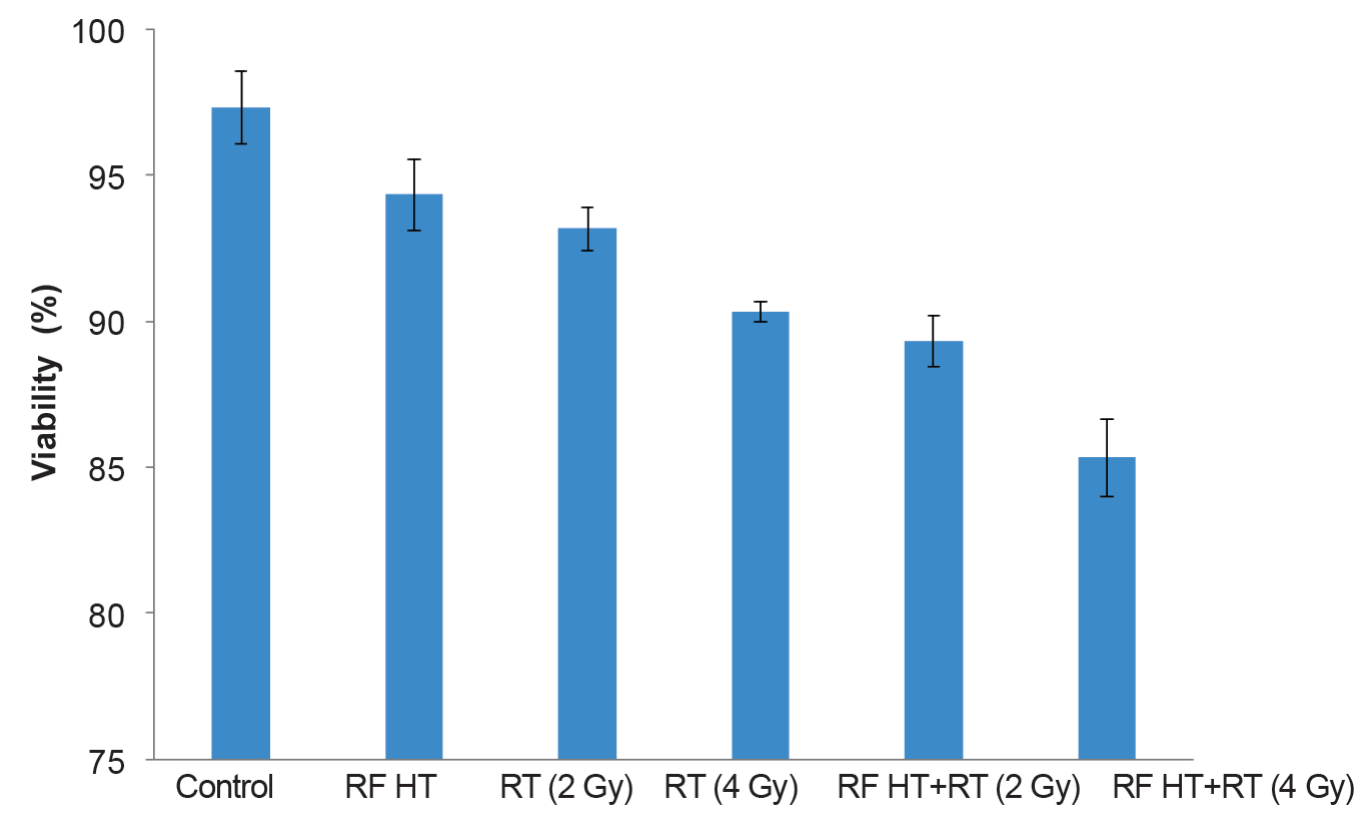
The effect of radio frequency hyperthermia+radiation therapy (RF HT+RT) on viability of DU 145 cells in spheroid cultures. We assayed cell viability approximately one hour after the treatments by using the trypan blue dye exclusion test as described in the materials and methods section (means ± SEM of three experiments).

The treatments had no significant effect on cell viability. Figure 3 shows the fraction of viable cells after every treatment. RF HT+RT (4 Gy) treatment showed the minimum viability. 

### Effect of treatments on DNA damage assayed by the alkaline comet assay

**Fig.4:**
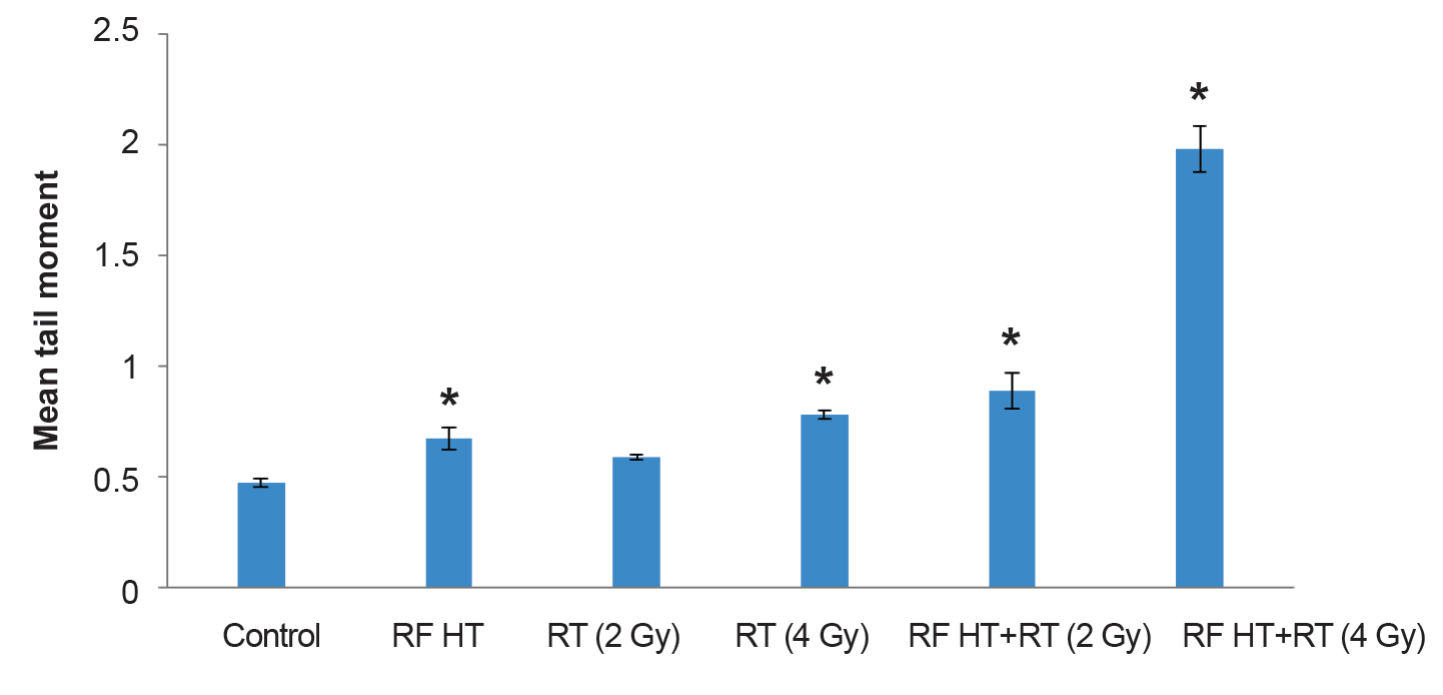
The amount of tail moments of spheroid cultures of DU 145 cells treated with radiofrequency hyperthermia (RF HT), radiation therapy (RT), and RF HT+RT. Spheroids exposed to 15 MV photons at 2 or 4 Gy doses and RF HT, (Celsius TCS system) for 90 minutes at 43˚C. Single cells were analyzed for DNA strand breaks. Tail moment, an evaluation of DNA strand breaks were determined by the alkaline comet assay. The data obtained for tail moment were analyzed statistically. *; Significant difference compared to the control group at P<0.05 (means ± SEM of three experiments).

We counted single cells and used 10^4^ cells for the alkaline comet assay. The average of tail moments (the product of tail length and the amount of DNA in the tail region) was introduced as an indication of DNA damage. The results (Fig.4) showed that DNA damages increased along with increased radiation dose. No significant difference existed in tail moments in the 2 Gy radiated groups compared with the control group. We observed significantly greater DNA damage in the RF HT+RT groups compared to the groups which received RT alone. Tail moment for RF HT alone showed a significant difference compared with the control group. Figure 4 shows that the tail moment had the highest increase with RF HT+RT (4 Gy) treatment. A method of examining the radiosensitizing effect of RF HT shown in Table 1 is to compare the levels of tail moment achieved for a combination of RF HT and RT with radiation alone. The ratio of the two tail moments is defined as TEF. Table 1 shows a TEF of 1.48 for the 2 Gy group and 2.54 for the group that received the 4 Gy radiation dose. The radiosensitizing effect of RF HT was more prominent after the 4 Gy radiation dose. 

### Effect of combined radiofrequency hyperthermia and radiation therapy on DU145 cell apoptosis 

**Fig.5:**
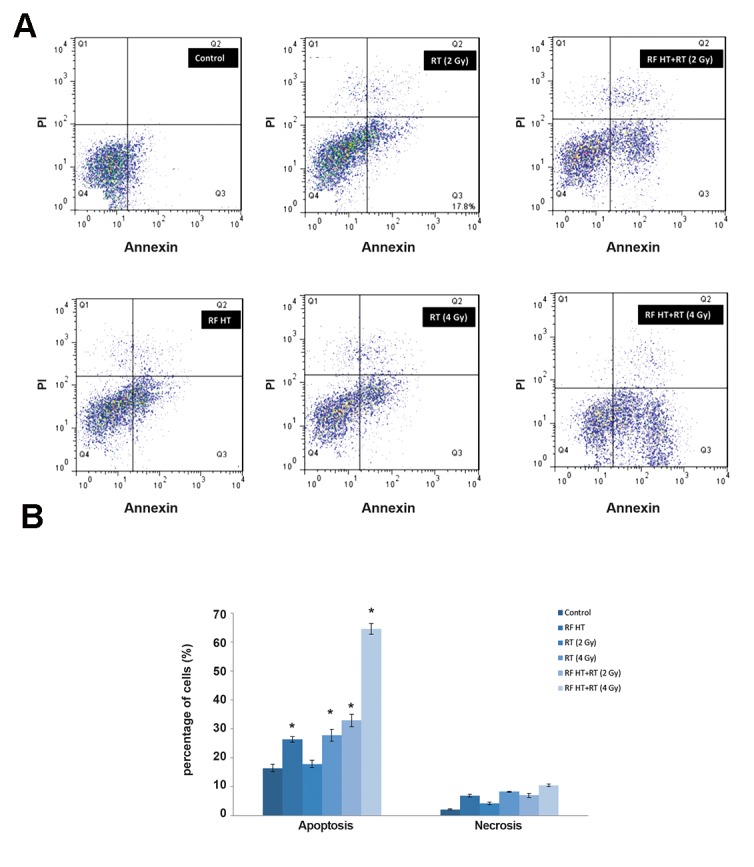
Analysis of apoptosis and necrosis in DU145 cells. A. BD FACSCalibur analysis of the apoptotic and necrotic cells using annexin V/PI one hour after cells were either exposed to radiofrequency hyperthermia (RF HT) at 43˚C for 90 minutes and radiation (RT) at 2 or 4 Gy of 15 MV X-rays; or the combination of HT and radiation (RF HT+RT) at 2Gy or 4 Gy. Annexin V/PI staining with FacScan dot plot analysis were used to divide the treated and control cells into four groups: Q1: percentage of necrotic cells, Q2: percentage of late apoptotic cells, Q3: percentage of early apoptotic cells, and Q4: percentage of living cells and B. Statistical analysis of percentage of apoptosis and necrosis of human prostate tumor cell line, DU 145, one hour after RF HT, RT, RF HT+RT (2 Gy), and RF HT+RT (4 Gy). Statistical analysis of the data obtained from flow cytometry analysis showed significant differences compared to the control group, marked with * (P<0.05) (means ± SEM of three experiments).

We used the DU145 prostate cancer cell line to determine the effect of combined RF HT and RT on the induction of apoptosis. This cell line was treated with RF HT followed by X-rays at either the 2 or 4 Gy dose administered at 15 minutes intervals. Annexin V/PI staining quantitatively determined the percentage of apoptosis and necrosis in these cells. As shown in Figure 5A, we divided the cells into four groups: living (Q4), early apoptotic (Q3), late apoptotic (Q2), and necrotic (Q1). The sum of cells in early apoptosis and those in late apoptosis were considered apoptotic cells (Q3+Q2). Necrotic cells were designated as the sum of those cells located in the upper left quadrant of the dot plot plus the late apoptotic cells (Q1+Q2). 

The untreated (control) samples mostly consisted of viable cells (84.6%) in addition to non-significant apoptotic and necrotic cells. Heat treatment enhanced the numbers of apoptotic cells compared to the control sample. There was an increase in apoptotic cell populations from unheated (15.3 ± 1.23%) to heated (26.3 ± 0.92%) cells. We observed increased numbers of apoptotic cells in the irradiated populations compared to the control population. The 4 Gy dose had higher numbers of apoptotic cells (27.7 ± 3.50) compared to the 2 Gy dose (17.8 ± 2.20). At the 2 Gy dose, no significant difference existed between the fraction of apoptotic cells and the control group, whereas we observed a significant increase in the percentage of apoptotic cells at the 4 Gy dose compared with the control group. The percentage of apoptotic cells after RF HT+RT at the 2 Gy dose was 32.9 ± 3.70%; whereas RF HT+RT (4 Gy) resulted in 64.48 ± 3.40% apoptotic cells. Both groups had significant differences compared to RT alone and the control group. The results indicated that RF HT+RT (4 Gy) treatment had the highest fraction of apoptotic cells (Fig.5). No significant fraction of necrosis existed after the treatments. The radiosensitizing effect of RF HT which achieved from annexin V/PI output is shown in Table 2 for a given combination of RF HT and RT with radiation alone. We considered TEF to be the ratio of two percentages of apoptotic cells. In Table 2, analysis after radiation doses of 2 Gy and 4 G resulted in a TEF of 1.85 for the 2 Gy dose and 2.33 for the 4 Gy dose. RF HT showed a more prominent effect after 4 Gy irradiation. 

**Table 1:** Thermal enhancement factor (TEF) from the comet assay for heated DU145 spheroids exposed to 15 MV photons at 2 and 4 Gy radiation doses


Dose (Gy)	Mean tail moment*		TEF^**^
	RT alone	RF HT+RT		

2	0.60 ± 0.02	0.89 ± 0.14	1.48
4	0.78 ± 0.03	1.98 ± 0.18	2.54


RF HT; Radiofrequency hyperthermia, RT; Radiation therapy, *; Mean tail moment values in this table were obtained from data shown in Figure 4, and **; Mean tail moment after RF HT+RT divided by mean tail moment after RT alone.

**Table 2:** Thermal enhancement factor (TEF) according to annexin V/PI flow cytometry for DU145 spheroids after treatment with radiofrequency hyperthermia (RF HT)+radiation therapy (RT) at 2 Gy and 4 Gy doses


Dose (Gy)	Mean tail moment*		TEF^**^
	RT alone	RF HT+RT		

2	17.80 ± 2.20	32.9 ± 3.7	1.85
4	27.70 ± 3.5	64.48 ± 3.4	2.33


*; Mean percentage of apoptotic cells values in this table were obtained from data shown in Figure 5B and **; Mean percentage of apoptotic cells after RF HT+RT divided by the mean percentage of apoptotic cells after RT alone.

### Discussion

We studied the toxicity and DNA damaging effects of RF HT (90 minutes at 43˚C) added to ionizing RT (15 MV X-rays) at two different radiation doses on spheroid cultures of DU145 cells. DU145 has been shown to establish large, stable spheroids in the liquid overlay technique (14). Multicellular spheroid cells represent a three-dimentional model similar to the tumor structure where cells are exposed to non-uniform distribution of oxygen and nutrients. In this study, we have used 300 µm diameter spheroids with a large hypoxic area. Hypoxic cells are sensitive to HT (7). HT at 43˚C leads to cell death mainly through the induction of lethal protein denaturation (15,16). Heat is not capable of causing serious DNA damage; it has the potential to block the repair of radiation-induced sublethal damage (SLD). Therefore, by intensifying radiation, DNA breaks are induced by denaturation of DNA repair enzymes (7). The combination of RF HT and ionizing radiation has been applied as a modality for treatment of locally advanced prostate cancer (17). A Celsius TCS capacitive system for loco- regional deep RF HT is available in some clinics. This system is a therapy system for the treatment of solid tumors, mostly used in combination with chemo- and/or radiotherapy in order to optimize the effects of these treatments (18). 

We applied RF HT before RT because *in vitro* and *in vivo* studies consistently showed that the application of RF HT before radiation resulted in remarkable enhancement of the radiation effect (19). The alkaline comet assay (single-cell gel electrophoresis) is reported to be the most sensitive method to assay DNA damage in individual cells. Under alkaline conditions (pH>13), this assay detects single-strand breaks (SSB) and double- strand breaks (DSB), in addition to excision repair and alkaline-labile sites (20). The cells are lysed in agarose. Following alkaline electrophoresis, the DNA strands migrate toward the anode, generating comet-like structures in the gel (referred to as a comet tail). The extent of migration depends on the number of strand breaks in the nucleoid. The migration is visualized and scored in a fluorescence microscope after staining. Apoptosis is a process of programmed cell death vigorously induced by the cell itself to provide for normal status. Necrosis, defined as cell death that results from extrinsic acute damage and an oxygen deprived cellular environment, differs from apoptosis. Signal transduction processes induce apoptosis in response to DNA damage induced by chemical agents and ionizing radiation through mechanisms that involve the p53 gene and caspase-3 activation (7). The annexin V/PI protocol is an approach used to discover apoptotic and necrotic cells through differences in plasma membrane integrity and permeability (21-23). 

In this study, the trypan blue exclusion assay showed no significant loss of cell viability after the treatments. Hence, we did not observe any instantaneous treatment-related fatal effects. 

According to the comet assay results, by adding RF HT to RT, we observed a significant increase in tail moment, which indicated increased DNA damage. The results showed that the RF HT+RT, with an increased radiation dose from 2 to 4 Gy resulted in a large increase in tail moment. Flow cytometry results indicated that the addition of heat increased radiation-induced apoptosis. The fraction of apoptotic cells increased with the increase in radiation dose. RF exposure does not appear to induce apoptosis. Most studies imply that the non-thermal effect of RF exposure does not cause breakage of DNA bonds (24). Koshkina et al. (25) have stated that the non-thermal effect of RF waves at 13.56 MHz in cancer cells induced autophagy but not apoptosis. These RF effects were absent in normal cells. According to these findings, it could be concluded that the enhanced fraction of apoptosis in RF HT and combination therapy seen in this study was due to the effect of heat generated from RF capacitive system. 

This data demonstrated that treatment with RF HT+RT would have a much greater effect on DNA damage and apoptosis induction in DU145 cells than radiation or RF HT alone. A number of studies have explained the cause of enhanced DNA damage and induced apoptosis observed after treatments in this study. Olive stated that DSBs could result in permanent cell cycle arrest, induction of apoptosis, or mitotic cell death caused by loss of genomic material (26). Charles and Rehm (27) observed that DNA damage, as induced by ionizing radiation and genotoxic chemotherapy, was a prototype inducer of intrinsic apoptosis. According to these findings, it could be concluded that enhanced DNA damage leads to apoptosis and vice versa. In addition, RF HT alone induces apoptosis and radiation alone induces DNA damages. The enhanced fraction of apoptosis from RF HT+RT in this study compared to RT alone might represent the fact that RF HT could inhibit the recovery of DNA damage and lead to apoptosis (7). The data for TEF presented herein demonstrated that DU145 human prostate carcinoma cells were radiosensitized by RF HT, which completely agreed with the results observed in previous studies that researched other types of HT (28,19). 

## Conclusion

We used the trypan blue exclusion assay, alkaline comet assay, and annexin V/PI flow cytometry to measure the viability, amount of DNA damage in individual cells as tail moment, and percentages of apoptotic cells in response to combined RF HT with RT (15 MV X-rays). In RF HT+RT, the addition of heat increased the radiation tail moments by a factor of 1.48 at the 2 Gy dose and 2.54 at the 4 Gy dose. In this group, radiation-induced apoptosis increased by a factor of 1.85 for the 2 Gy radiation dose and 2.33 for the 4 Gy radiation dose. We observed a thermally enhanced dose response in the DU145 prostate cancer cell line with RF HT combined with mega voltage external X-rays. The extent of enhancement depended on the radiation dose. RF HT could be effective as a synergetic treatment in cancer radiotherapy because of the ability to provide lower doses of radiation, especially for androgen independent prostate cancer cells such as DU145. 
